# Psychedelic‐assisted therapy for functional neurological disorders: A theoretical framework and review of prior reports

**DOI:** 10.1002/prp2.688

**Published:** 2020-12-05

**Authors:** Benjamin Stewart, Jon G. Dean, Adriana Koek, Jason Chua, Rafael Wabl, Kayla Martin, Naveed Davoodian, Christopher Becker, Mai Himedan, Amanda Kim, Roger Albin, Kelvin L. Chou, Vikas Kotagal

**Affiliations:** ^1^ Department of Neurology University of Michigan Ann Arbor MI USA; ^2^ Department of Molecular and Integrative Physiology University of Michigan Ann Arbor MI USA; ^3^ National Herbarium of Victoria Melbourne VIC Australia; ^4^ University of Chicago Pritzker School of Medicine Chicago IL USA; ^5^ Present address: Saint Alphonsus Medical Group Neurology Boise ID USA; ^6^ Present address: Department of Neurology University of Washington Seattle WA USA; ^7^ Present address: Department of Psychiatry Brigham and Women's Hospital Harvard Medical School Boston MA USA

**Keywords:** conversion disorder, hallucinogens, hysteria, psychosomatic medicine, receptor, serotonin, 5‐HT2A

## Abstract

Functional neurological disorders (FNDs), which are sometimes also referred to as psychogenic neurological disorders or conversion disorder, are common disabling neuropsychiatric disorders with limited treatment options. FNDs can present with sensory and/or motor symptoms, and, though they may mimic other neurological conditions, they are thought to occur via mechanisms other than those related to identifiable structural neuropathology and, in many cases, appear to be triggered and sustained by recognizable psychological factors. There is intriguing preliminary evidence to support the use of psychedelic‐assisted therapy in a growing number of psychiatric illnesses, including FNDs. We review the theoretical arguments for and against exploring psychedelic‐assisted therapy as a treatment for FNDs. We also provide an in‐depth discussion of prior published cases detailing the use of psychedelics for psychosomatic conditions, analyzing therapeutic outcomes from a contemporary neuroscientific vantage as informed by several recent neuroimaging studies on psychedelics and FNDs.

**Abbreviations**
5‐HT2A: Serotonin 2A
ALD‐52: 1‐acetyl LSD
BOLD: Blood‐oxygen‐level‐dependent
CBT: Cognitive Behavioral Therapy
CZ‐74: 4‐Hydroxy‐N,N diethyltryptamine
DMN: Default Mode Network
dmPFC: Dorsal Medial Prefrontal Cortex
DMT: N, N‐Dimethyltryptamine
FND: Functional Neurological Disorder
FNSD: Functional Neurological Symptom Disorder
IM: Intramuscular
LSD: Lysergic Acid Diethylamide
MLD‐41: 1‐methyl LSD
OCD: Obsessive Compulsive Disorder
PCC: Posterior Cingulate Cortex
PNES: Psychogenic Nonepileptic Spells
REBUS: Relaxed Beliefs Under Psychedelics
RSFC: Resting State Functional Connectivity
TPJ: Temporoparietal Junction
vmPC: Ventral Medial Prefrontal Cortex

## 1 INTRODUCTION

A resurgence of interest in psychedelic research^1^, ^2^ has led to the conceptual consideration of psychedelic‐assisted psychotherapy for a growing number of neurological and psychiatric disorders.^2^, ^3^

In their important 2017 article, Bryson et al argued that psychedelics may be useful in the treatment of functional neurological disorders (FNDs). They describe changes in hierarchical brain dynamics and specific functional brain networks in response to 5‐HT2A agonists (classical psychedelics) and compare these with alterations in brain function thought to occur in patients with FNDs.^4^

In the text that follow, we explore and expand upon this theoretical framework. We also include a review of relevant case reports and case series as an empirical counterpart to these conjectural propositions.

## 2 OVERVIEW OF FUNCTIONAL NEUROLOGICAL DISORDERS

While FNDs are not new within the field of neurology, the conceptions of these illnesses, as well as the terminology used to describe them, have evolved substantially over the course of history. Symptoms that would now be classified as functional have previously been attributed to causes ranging from the supernatural to the direct and indirect manifestations of uterine disease. While the term "hysteria" has persisted in modern medical parlance to some extent, by the late 17th century, uterocentric theories of these disorders began to give way to explanations implicating the brain.^5^

Between the late 19th and early 20th centuries, considerable effort was devoted to classifying and understanding these disorders, with significant contributions made by prominent neurologists including Pierre Briquet and Jean Martin Charcot.^5^, ^6^ However, the insights of one of Charcot's students, Sigmund Freud, have yielded perhaps the most enduring impact on our conceptions of functional neurological symptoms. Freud coined the term "conversion" in 1894, referencing his belief that “hysterical” symptoms resulted when intrapsychic conflict was transformed into somatic manifestations.^7^ While Freud's ideas are not without their detractors, the term "conversion disorder" remains listed in the most recent edition of the Diagnostic and Statistical Manual of Mental Disorders (DSM‐5) as synonymous with the newer terminology of functional neurological symptom disorder (FNSD).^8^

In contrast to the specific language of the DSM‐5, the informal labels given to medically unexplained symptoms remain somewhat heterogeneous. While “psychogenic,” “psychosomatic,” and “nonorganic” all remain in use, there is growing preference for the word “functional”.^9^ Within the field of neurology, the term FND is more commonly used than FNSD or conversion disorder, and additional diagnostic labeling is often used to distinguish among different presentations. For example, functional motor disturbances can be referred to as functional movement disorders and can be further subdivided into functional gait disturbance, tremor, dystonia, myoclonus, chorea, and weakness.^10^ Other presentations include functional blindness, aphonia/dysphonia, and sensory changes. Functional seizures are often referred to as psychogenic nonepileptic spells (PNES).^9^ Whether or not these phenotypes represent biologically distinct entities or different manifestations of the same underlying process remains a matter of some controversy, though that individual patients often display multiple types of functional symptoms has been cited by some as evidence of the latter.^11^

Historically, FNDs have carried a generally poor prognosis.^12^, ^13^, ^14^, ^15^ A 2014 systematic review that included over 2000 patients with functional movement disorders found that only 20% of patients experienced complete remission of symptoms, while 40% had similar or worse symptoms to their initial presentation at long‐term follow‐up.^16^ PNES are associated with similarly poor outcomes, with more than half of patients continuing to have episodes at the time of follow‐up.^12^, ^14^ The significance of the low recovery rates seen in FNDs is underscored by the impact these disorders have on patients. For example, two separate studies found patients with functional movement disorders to have similar or greater reductions in quality of life to patients with “organic” movement disorders.^17^, ^18^ A recent study also found that patients with PNES have a standardized mortality ratio that is 2.5 times higher than the general population, similar to that of patients with medically refractory epilepsy.^19^

Recent research into effective treatment practices may help reshape the prognosis of functional disorders. In the case of functional movement disorders, there is increasing evidence to support modified intensive physical rehabilitation programs.^10^, ^20^, ^21^ One such study based out of the Mayo Clinic reported that upon completion of a week‐long outpatient program, nearly three quarters of patients demonstrated at least marked improvement.^20^ Similarly, Lafrance et al^22^ reported that a 12‐week cognitive behavioral therapy (CBT)‐based program for PNES resulted in a reduction of spell frequency by approximately 50%.

While the results of such programs are encouraging, treatment responses are often incomplete, and many patients fail to derive any significant benefit. For example, in the aforementioned Mayo Clinic study, at long‐term follow‐up, over 60% of patients continued to report persistent symptoms, and 25% reported little to no improvement from baseline.^20^ Furthermore, issues of access and cost of treatment associated with such programs limit their practical utility and argue the need for other interventions. While benefit has been reported with other therapeutic strategies, including sedative‐hypnotics and transcranial magnetic stimulation, studies to date have been small, and further research is needed.^23^

## 3 OVERVIEW OF THE HISTORY AND PHARMACOLOGY OF PSYCHEDELIC COMPOUNDS

Grinspoon and Bakalar describe a psychedelic substance as one that, “without causing physical addiction [or] major physiological disturbances […] produces thought, mood, and perceptual changes otherwise rarely experienced except in dreams, contemplative and religious exaltation, flashes of vivid involuntary memory, and acute psychosis”.^24^, ^25^

Human experiences with psychedelics long predate knowledge of their molecular nature. Among the earliest archeologically documented consumers of psychedelic agents were aboriginal communities of northeastern Mexico and Trans‐Pecos Texas.^26^ Radiocarbon dating demonstrates that these communities used peyote, *Lophophora williamsii*, the mescal bean *Sophora secundiflora*, and the Mexican buckeye *Ungnadia speciosa* as far back as circa 8500 BCE.^27^ Alongside plants, fungi are also natural sources of psychedelic chemicals, psilocybin‐containing mushrooms being among the best‐known examples. Such mushrooms are of major significance to indigenous traditions associated with Mesoamerica.^28^

The unifying pharmacological property of the classical psychedelics appears to be agonism of serotonin 2A (5‐HT2A) receptors.^29^ Elucidation of this mechanism began with the discovery of serotonin in the mammalian brain in the 1950s^30^ and recognition of the structural similarity between serotonin and lysergic acid diethylamide (LSD).^31^ While the exact interaction between psychedelics and the serotonergic system was unclear for some time, the development of subtype‐specific serotonin antagonists narrowed focus on the 5‐HT2A receptor, the blockade of which was found to inhibit behavioral effects of various psychedelics in rats and, in later studies, to block the effects of psilocybin and LSD on subjective perceptual experience in humans.^3^, ^32^, ^33^, ^34^, ^35^

A key step in inaugurating the study of psychedelic compounds in academic research was the synthesis of LSD by Swiss chemist Albert Hofmann in 1938. Though initially labeled as being of no particular interest by the pharmacology division at Sandoz Laboratories, Hoffman resynthesized LSD in 1943, inoculating himself with the molecule in the process by accident. This experience, along with a purposeful ingestion a few days later, revealed the mind‐altering effects of LSD and provided the first hints that it may be of relevance to the study and treatment of psychiatric disease.^36^

Early studies of LSD in human subjects yielded little success in treating psychotic disorders but demonstrated exciting potential for the treatment of a variety of nonpsychotic mental illnesses including mood disorders and addiction.^2^, ^37^, ^38^, ^39^ Despite this promise, passage of the Controlled Substance Act of 1970 presented a legal barrier to scientific investigation involving psychedelic substances and an ongoing challenge to reintroducing their status as tools in research and therapy.^3^, ^24^, ^40^, ^41^

Psychedelic research stagnated for two decades until studies reporting the neurobiological correlates of psilocybin, DMT, and mescaline emerged in the 1990s, followed by new studies evaluating their therapeutic potential in certain psychiatric disorders.^2^, ^42^, ^43^, ^44^, ^45^ These initial studies, together with efforts to formally reintroduce psychedelics as instruments of science and medicine by professional organizations (eg, the Multidisciplinary Association for Psychedelic Studies), galvanized a resurgence in psychedelic research.^46^, ^47^

In recent decades, an increasingly robust body of literature has emerged supporting the safety and therapeutic value of psychedelic substances in a variety of mental health contexts.^2^, ^3^ Three separate studies of psilocybin‐assisted psychotherapy demonstrated posttreatment improvement of depression and anxiety in patients with life‐threatening cancer. In the two larger studies, continued benefit was seen in 60%‐80% of patients at 6 months^48^, ^49^, ^50^; a recent follow‐up to one of those studies found effects to be sustained at 4.5 years.^51^ Promising preliminary results have also been observed with psilocybin‐assisted psychotherapy in alcoholism,^52^ smoking cessation,^53^ obsessive compulsive disorder,^54^and treatment‐refractory major depression.^55^, ^56^ There is interest in psychedelic‐assisted therapy as a potential treatment for a number of other disorders including anorexia nervosa,^57^ bipolar disorder,^58^ chronic pain,^59^ and cocaine addiction.^60^

Some feel the introduction of psychedelic‐assisted psychotherapy marks the beginning of a new era in the field of psychiatry. However, it remains a topic of debate whether the subjective changes in consciousness induced by psychedelics can be dissociated from the underlying biological changes with regard to their therapeutic efficacy. Whereas the therapeutic effects of traditional psychopharmaceuticals are considered to be a direct result of changes in neurotransmitter levels and downstream signaling pathways in the context of continued dosing,^61^ psychedelics may be capable of producing benefits that long outlast their direct pharmacological effects by facilitating context‐dependent changes in cognitive and emotional processing during the acute drug state.^62^

## 4 MECHANISTIC MODELS OF FNDS AND THEIR RELEVANCE TO PSYCHEDELIC AGENTS

### 4.1 The default mode network and neurobiological correlates of Freudian conceptions

Among the network changes induced by psychedelics, much attention has been given to their effect on the default mode network (DMN). The DMN is comprised of functionally connected brain regions, including the ventral medial prefrontal cortex (vmPFC), dorsal medial prefrontal cortex (dmPFC), posterior cingulate cortex (PCC), precuneus, and lateral parietal cortex,^63^ and is most active when individuals are not engaged in goal‐directed tasks.^63^, ^64^, ^65^, ^66^

The DMN appears to be important in self‐related processing,^4^, ^67^ and it has been argued to correspond to Freudian conceptions of the ego.^67^, ^68^, ^69^ The 5‐HT2A receptor is densely expressed throughout the DMN,^70^, ^71^ and fMRI studies have shown that psychedelic agents acutely suppress blood‐oxygen‐level‐dependent (BOLD) signal in key nodes of this network and also suppress its within‐network resting state functional connectivity (RSFC).^72^, ^73^, ^74^ This may explain some of the subjective effects of psychedelics, including feelings of increased connectedness and, at high doses, a phenomenon known as “ego death,” in which individuals describe a dissolution of sense of self.^3^, ^67^, ^73^, ^74^, ^75^ In his recently published book on psychedelics, Michael Pollan likens this experience to feeling like a pile of post‐it notes scattered to the wind, going on to say, “But the ‘I’ taking in this seeming catastrophe had no desire to chase after the slips and pile my old self back together […]. And then I looked and saw myself out there again, but this time spread over the landscape like paint, or butter, thinly coating a wide expanse of the world […]”.^76^

Many of the proposed therapeutic benefits of psychedelics have been theorized to relate to their action on the DMN.^74^ For example, depressive symptoms have been argued to result from the brain entering into an overly ruminative state, corresponding on a network level to pathologic increase of within‐network DMN functional connectivity^67^, ^75^, ^77^, ^78^. Interestingly, and in opposition to the acute effects of psychedelics on the DMN, Carhart‐Harris et al ^78^ found enhanced DMN integrity in a cohort of patients with refractory depression the day following treatment with psilocybin. This finding has led some to liken the therapeutic action of psychedelics to pressing a reset button for the brain.^78^, ^79^ This may explain the preliminary evidence that addiction, which has been associated with decreased baseline DMN RSFC,^25^ may also benefit from psychedelic‐assisted psychotherapy.^52^, ^53^

These findings are of relevance to proposed studies on psychedelic administration for treatment of FNDs. Several studies have reported abnormalities of DMN activity in patients with FNDs.^80^, ^81^, ^82^, ^83^ In a study of patients with functional seizures, Van der Kruijs et al ^83^ reported increased coactivation of the precuneus and cingulate gyri as compared to controls in the resting state and additionally found that, among patients, DMN RSFC strength was positively correlated with scores on dissociation questionnaires. Similarly, Monsa et al^82^ found enhanced DMN RSFC in seven patients with unilateral functional paresis. In another study of unilateral functional paresis, De Lang et al^81^ found failure of the vmPFC to deactivate during implicit motor imagery tasks corresponding to the affected limb. Cojan et al^80^ reported similar findings during motor preparation in a patient with functional left arm paralysis, additionally noting increased functional connectivity between the right motor cortex and various nodes of the DMN including the vmPFC, precuneus, and posterior cingulate cortex. The findings of increased within‐network DMN functional connectivity in patients with FNDs are of particular interest when considering psychedelic therapy as a potential treatment for these disorders. Specifically, the ability of psychedelics to acutely diminish RSFC between nodes of the DMN^72^, ^73^, ^84^, ^85^, ^86^, ^87^ could conceivably represent a biological correlate of the amelioration of FND symptoms induced by psychedelic administration. Examples of the latter are outlined in the case reports section.

As mentioned previously, the DMN has been theorized to be the neurobiological correlate to the Freudian ego. In keeping with this model, increased DMN RSFC, and its downstream inhibition of limbic activity, has been proposed as the mechanistic underpinning for Freudian repression.^68^, ^69^ This may bear relevance to a number of conditions^68^, ^77^ but perhaps to FNDs in particular, in which repression of negative thoughts and emotions has remained central to generative models of symptoms^88^, ^89^, ^90^ since Freud first introduced the idea of conversion in 1894.^7^

Given the ability of 5‐HT2A agonists to suppress DMN functional connectivity, their ability to break down repressive barriers may come as no surprise. Indeed, this feature of psychedelics has long been recognized, with the pioneering psychedelic researcher Stanislov Grof calling LSD a “nonspecific amplifier of the unconscious”.^3^ Numerous studies describe the reemergence of unconscious material during psychedelic‐induced states of altered awareness.^91^, ^92^, ^93^, ^94^, ^95^, ^96^, ^97^, ^98^, ^99^, ^100^, ^101^, ^102^, ^103^, ^104^, ^105^ In a 1958 paper, Eisner and Cohen wrote “the recall and ‘reliving’ of past events is enhanced; frequently whole sequences unroll before the patient's eyes as though they had been stored on microfilm”.^96^ As discussed in the case reports section, such episodes of abreaction^*^ have been described in a number of patients with FNDs treated with psychedelics.^95^, ^97^, ^99^, ^103^

### 4.2 The temporoparietal junction and disruptions in the agency network

In addition to their effects on the DMN, psychedelics have been shown to acutely alter functional connectivity within and between various other brain regions.^86^, ^87^, ^107^, ^108^, ^109^

The ability of psychedelics to alter connectivity between brain regions may bear particular relevance to the treatment of functional movement disorders. In these disorders, while motor symptoms may have the appearance of voluntariness, patients experience movements as outside their volitional control—stated differently, there is an apparent loss of motor agency.^110^ While the agency network is broadly distributed, the right temporoparietal junction (TPJ) is thought to play a critical role.^110^, ^111^, ^112^ Specifically, the right TPJ is thought to be involved in mismatch detection between performed and intended movements.^111^ The TPJ is also thought to be important for maintenance of embodiment, and TPJ dysfunction has been implicated in the genesis of dissociative symptoms.^113^, ^114^

In a 2016 study, Mauer et al demonstrated impaired functional connectivity between the right TPJ and bilateral sensorimotor regions in patients with a variety of functional movement disorders.^111^ Similar results were seen in a 2010 study of functional tremor.^112^ In this way, functional movement disorders might be conceptualized as functional disconnection syndromes, in which motor networks have impaired communication with networks responsible for production of agency. Monsa et al^82^ reported other abnormalities in functional connectivity involving the TPJ in patients with unilateral functional limb weakness, finding decreased connectivity between the TPJ and medial temporal lobes and increased connectivity between the TPJ and the limbic/salience network. Other changes in functional connectivity, both involving and not involving the TPJ, have been noted in various studies,^80^, ^81^, ^115^ with significant heterogeneity in the findings from each group. Whether this heterogeneity relates to differences in patient population, protocol, processing techniques, or some combination thereof remains uncertain.

As for relevant changes in functional connectivity induced by psychedelic agents, and continuing with the TPJ as an example, Tagliazucchi et al^109^ found that IV infusion of LSD resulted in increased global brain connectivity of the bilateral TPJ, and that this was positively correlated with ego dissolution scores. Furthermore, in a subsequent seed‐based analysis, LSD infusion was found to acutely increase connectivity between the TPJ and sensorimotor cortex. Though an intriguing prospect, whether or not psychedelic agents could reliably reverse the baseline hypoconnectivity between TPJ and sensorimotor cortex in patients with functional movement disorders is unclear. It is further unclear whether such repairing of connectivity would result in symptomatic improvement.

As with functional imaging of patients with FNDs, the changes in connectivity reported to occur with administration of psychedelic agents vary significantly from study to study. For example, and in opposition to the findings of Tagliazucchi et al, Preller et al found that both LSD and psilocybin resulted in decreases in global connectivity of the TPJ.^86^, ^87^ In addition to uncertainty related to the acute alterations in functional connectivity induced by psychedelic agents, how these changes evolve over time remains largely unexplored.

While an in‐depth discussion of the various changes in brain networks and functional connectivity that occur with administration of 5‐HT2A agonists is beyond the scope of this paper, a recent review by Franz Vollenweider and Katrin Preller summarizes these and other neurobiological effects of psychedelic agents. The authors tie these changes to psychedelic‐induced alterations in perception, cognition, and behavior, and link these effects to hypothesized therapeutic concepts in several psychiatric conditions.

### 4.3 Suggestibility and Bayesian remodeling

As discussed in detail in the case reports section that follows, Edward Baker published a 1967 case report of a man with functional weakness of three limbs which resolved with LSD‐assisted psychotherapy. In the discussion of his report, Baker comments, “It is impossible to state how much was drama and suggestion vs how much LSD forcing of repressive barriers; we are awaiting more cases to arbitrate the point.”^91^

The power of suggestion has long been recognized to have both diagnostic and therapeutic value in functional disorders. This power of suggestion, combined with the poor prognosis and relative lack of other effective therapies, has led some to advocate for the ethical use of placebo in the treatment of FNDs.^116^, ^117^ Perhaps not surprisingly, there is evidence that hypnosis, a technique known for its ability to enhance suggestibility, has benefit in treating FND symptoms.^118^, ^119^ Charcot himself would often use hypnosis to induce and relieve functional symptoms in “hysteria” patients during his clinical lessons at La Salpêtrière.^120^ Similarly, a 2010 meta‐analysis of “drug interviews” (predominantly utilizing sedative‐hypnotics) for conversion disorder found suggestibility to be a positive predictor of recovery.^90^ It bears mentioning that, like psychedelics, hypnosis^121^, ^122^ and sedative‐hypnotics^123^ have been shown to have a suppressive effect on within‐network functional connectivity of the DMN.

The ability of psychedelics to increase suggestibility has been noted at least since early clinical work with LSD began in the 1950s^124^ and has been demonstrated empirically by at least three different studies.^124^, ^125^, ^126^ Possibly related is the observation that psychedelic experiences often carry a noetic quality,^25^, ^74^, ^79^, ^127^, ^128^ such that insights gained under the influence of psychedelics are sometimes perceived by subjects as more true/important than waking reality.^79^ These properties at once underscore the powerful therapeutic potential of psychedelics and portend their potential for harm if used incautiously.^77^

The importance of suggestibility in functional disorders may relate to the reshaping of inaccurate prior “expectations” or “beliefs.” Edwards et al^11^ proposed a Bayesian model of how FNDs develop and are maintained. The authors argue that functional symptoms are the result of errors in predictive processing, in which incorrect and overly precise prior expectations negate the influence of contradictory sensory input—essentially, top‐down processing overwhelming bottom‐up processing. More generally, they propose that perception is not so much a true reflection of the natural world but a compromise between the truth and what one expects the truth to be. They further point out that the ability of expectation to influence experience can be demonstrated in healthy individuals. In a 2005 study, Lorenz et al demonstrated that the perception of intensity of noxious stimuli was impacted by cuing prior to stimulus delivery. If subjects were cued that they would be receiving a strong stimulus, there was a tendency to rate the stimulus intensity higher than if they were told they would be receiving a weak stimulus; the reverse was also true. Interestingly, secondary somatosensory evoked potential amplitudes as measured by magnetoencephalography were similarly impacted by cuing.^129^ While the Bayesian explanation of functional sensory symptoms may be more intuitive, this model can be used to explain functional motor symptoms as well. The argument made by Edwards and colleagues^11^ is that the primary deficit underlying functional motor symptoms is one of the abnormal proprioceptive beliefs which are then fulfilled by a motor response or, in the case of functional weakness, a lack of motor response. In the proposed model, while abnormal beliefs about symptoms do not originate at the conscious level, conscious attention to the symptoms reinforces those abnormal beliefs; this may explain why functional symptoms often improve with distraction.^11^ The Bayesian model may also explain the finding that, in the treatment of functional motor disturbances, even modest improvements can promote more substantial gains,^130^ perhaps by forcing revision of aberrant prior beliefs.

Bayesian modeling has been used to explain other phenomena ranging from common optical illusions, to biases in worldview, to various psychiatric disease states such as depression and obsessive‐compulsive disorder.^77^ The basic premise is similar to the model for FNDs^11^: heavily weighted prior expectations lead to top‐down suppression of bottom‐up influences resulting in cognitive distortions that are not easily overcome by conflicting input.^67^, ^75^

**FIGURE 1:**
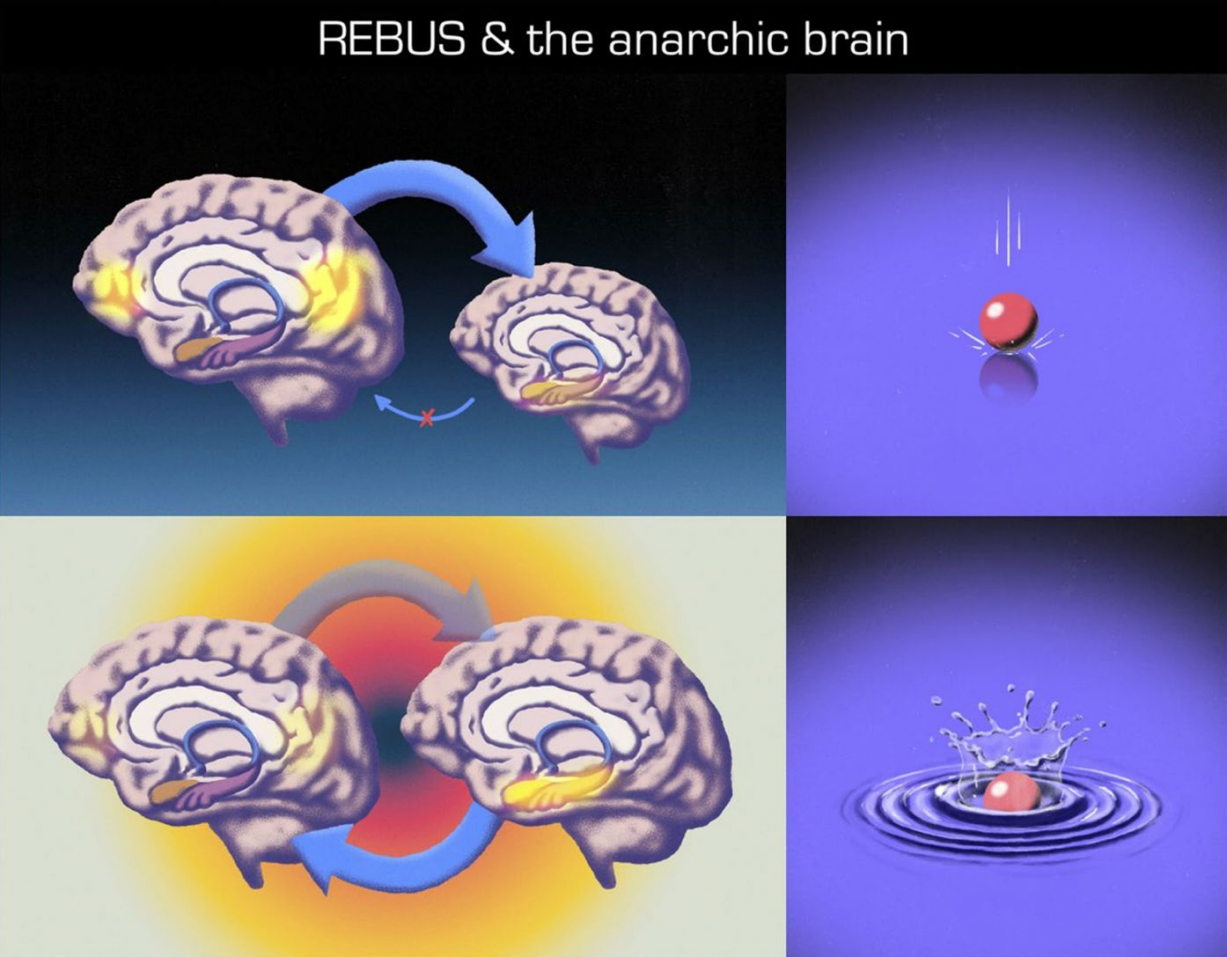
The top row depicts aberrant brain dynamics in which over‐weighted, high‐level priors overwhelm bottom‐up signalling, which is represented in the top left panel by the big and small arrows, respectively. These dynamics, thought to be common to many psychopathologies, create an overly rigid state in which the brain is relatively insensitive to ascending information. This is depicted by the ball in the top right panel landing without causing a surface perturbation. The bottom row depicts brain dynamics during psychedelic states. In the bottom left panel, the two brains are of equal size, intended to represent flattening of the Bayesian hierarchy. The increased translucence of the top‐down arrow represents de‐weighting of high‐level priors, and the increase in thickness of the bottom‐up arrow reflects increased influence of ascending information, such as from the limbic system. This increased sensitivity to bottom‐up signalling is represented in the bottom right panel; the same ball now creates ripples on a less rigid surface. Figure reproduced with permission from the article REBUS & and the anarchic brain by Carhart‐Harris and Friston.^77^ Illustrations by Pedro Oliveira, courtesy of Favo Studio

Building on prior work,^67^, ^131^, ^132^ Robin Carhart‐Harris and Karl Friston have argued^77^ that psychedelics may carry a unique ability to temporarily flatten the brain's Bayesian hierarchy, providing a window during which aberrant prior expectations are softened and can be recalibrated (Figure 1). The authors cite the dense expression of 5‐HT2A receptors in association cortex as well as functional imaging and electrophysiologic data to support this schema which they have termed REBUS (relaxed beliefs under psychedelics). At a behavioral/experiential level, this formulation of psychedelic action may be more intuitively understood. Carhart‐Harris and Friston provide the example of the commonly reported psychedelic‐induced visual illusion of seeing the walls “breathe.” They argue that under normal circumstances, the heavily weighted prior expectation that walls are static prevents the incomplete fidelity of ascending visual information from being interpreted as motion, but that, in the presence of 5‐HT2A agonism, these top‐down refinements are lost.^77^ Another example is the hollow mask illusion in which a rotating plain mask is perceived as forward facing whether the viewer is looking at it from the front or the back.^133^ Dissolution of this phenomenon has been reported to occur under psychedelic states.^134^

While this review uses terminology such as “beliefs” and “expectations” in reference to high‐level priors, this is not meant to imply that these are volitionally formed or maintained at a conscious level. Conversely, in some cases, as exemplified by the hollow mask illusion, high‐level priors may directly oppose conscious understanding. This is often the case in FNDs, in which patients may come to understand the nature of their symptoms on a conscious level, but that understanding does not necessarily lead to symptom amelioration. This supports the idea, as suggested by Edwards et al, that the primary disturbance in FNDs occurs at some intermediate level of the Bayesian hierarchy.^11^

Therefore, what has to this point been referred to as expectations and beliefs would perhaps be better described as high‐level constructs encoded by neural tissue. An illustrative example of this is the phenomenon of phantom limb sensations, in which patients with amputations, despite understanding at a conscious level that a limb is gone, feel as though it is still present. Interestingly, alleviation of phantom limb pain through the use of psychedelics has been described in the literature.^135^, ^136^, ^137^, ^138^ Ramachandran et al detailed the case of a 35‐year‐old man with phantom right leg pain failing to respond satisfactorily to mirror therapy.^†^ The patient noted partial and transient relief of his symptoms after taking psilocybin‐containing mushrooms, only to have his symptoms resume their prior intensity once the acute drug effects had worn off. However, when the patient tried mirror therapy while still in a psychedelic state, he experienced more substantial and longer‐lasting effects and was eventually able to discontinue treatment altogether.^138^ The improvement the patient experienced with psychedelic‐assisted mirror therapy, as well as the unsatisfactory response with psychedelic administration alone, is explained by the REBUS model. That is to say, while psychedelics may make high‐level constructs more amenable to reshaping, the appropriate bottom‐up influences must be present for this reshaping to occur in a therapeutic way.

It should be noted that the Freudian framework involving the DMN and its suppression of limbic activity detailed in subsection 4.1 is concordant with the Bayesian brain model.^67^, ^77^ Carhart‐Harris and Friston propose that the DMN is situated at the top of the brain's functional hierarchy and exerts top‐down inhibition of ascending information including, and importantly, from intrinsic sources such as the limbic system.^77^

## 5 CASE SERIES AND REPORTS OF PSYCHEDELIC THERAPY FOR FUNCTIONAL NEUROLOGICAL DISORDERS

A number of case series from the pre‐prohibition era of psychedelic research include patients labeled as “conversion,” “hysteria,” and “psychosomatic”^140^ (Table 1). However, in the majority of these papers, no details of the patients’ presentations are given, making it difficult to know whether their diagnoses would be classified as functional by modern standards. For instance, in a 1958 case series, Betty Eisner and Sidney Cohen refer to constipation and dysmenorrhea as psychosomatic illnesses,^96^ with other authors applying this terminology to such varied symptoms as headache, dizziness, numbness, back pain, stomach trouble, asthma, dermatitis, and allergic rhinitis.^105^, ^141^ In a 1959 report, Margot Cutner uses the term hysteria to refer both to conversion symptoms as well as histrionic tendencies, describing one “hysteric” patient as having primary symptoms of “sporadic depressions, various fears and uncontrollable tempers.” Elsewhere in the series, Cutner describes a patient with generalized stiffness, speech changes, and a feeling of “deadness” affecting the left‐hemibody that resolved with LSD‐assisted psychotherapy, but did not attempt to classify the symptoms as being part of a broader disorder.^94^

**TABLE 1:** Case Series and reports

Study	Substance	Dose	# of sessions	Scale	Remarks
Busch and Johnson (1950)^92^	PO LSD	Average dose 30‐40 mcg	Limited details	No scale provided. See remarks	Includes three patients of interest: A patient with "psychoneurosis hysteria" was documented to relive traumatic childhood events whereas previous amytal interviews had failed. A patient labeled as "psychosomatic" was noted to relive a disturbing navy experience whereas prior attempts of narcoanalysis were only partially successful; the patient improved enough to be able to discontinue therapy. A patient with "psychoneurosis neurasthenia" became "more disturbed but better able to discuss problems."
Anderson and Rawnsley (1954)^142^	PO LSD	30‐600 mcg	Average of 2.5 per patient	No scale provided. See remarks	Includes four patients with "hysteria." Only one of these is described. The author states, "an inadequate psychopath with psychogenic amnesia felt much better in respect of increased self‐confidence and hope for the future following a total of 1500 gamma LSD in 4 doses, but the drug did not help in restoring his memory." Following discharge the patient was lost to follow‐up.
Sandison et al (1954/1957)^104^, ^143^	LSD (route unspecified)	Starting dose 25 mcg. Limited information regarding escalation. One patient received 400 mcg	Limited details	Recovered Greatly Improved Moderately Improved Improved Not Improved	This series included 4 patients with "conversion hysteria," 1 who was listed as recovered, 1 as moderately improved, 1 as improved, and 1 who was not classified because the patient refused further treatment after a single session. Sandison and Whitelaw published a 1957 extension of the 1954 series in which they reported the results of patients with "hysteria (all forms)" rather than the previous classification of "conversion hysteria." In this latter series, they rate 1/12 as recovered, 2/12 as greatly improved, 4/12 as moderately improved, and 5/12 as not improved. The classification "not improved" was used if improvement was not sufficient to allow full return to work. While information on dosing was limited, in a 2018 review by Rucker et al,^2^ the approach used by Sandison was described as "psycholytic."
Eisner and Cohen (1958)^96^	LSD (route unspecified); in a few cases, ALD‐52^a^ or MLD‐41^b^ was given	Starting dose of LSD 25‐50 mcg. Escalation as high as 250 mcg	1‐16; average of 4.6 per patient	Patients listed as either improved or unimproved based on consensus of the therapist, patient, and a close contact of the patient. See remarks	Includes a 31 year old male patient labeled as "conversion reaction in a passive‐aggressive personality." No details of his presentation are given, but he was treated with 5 sessions of LSD (25, 50, 75, 100, and 150 mcg) as well as a single 500 mcg dose of ALD and was classified as improved. The series also includes two patients labeled as having "hysterical personality" who were treated with LSD, one of whom improved and one who did not.
Cutner (1959)^94^	PO LSD	25‐400 mcg	Limited details	No scale provided. See Remarks	Includes two cases of interest. Though not given a diagnostic label, Cutner describes the case of man with various symptoms including general rigidity, speech changes, and a feeling of deadness affecting the left hemibody. Symptoms resolved following an unspecified number of LSD‐assisted psychotherapy sessions. Additionally described is a case of a "hysteric," a middle aged woman with multiple symptoms including issues with temper and "psychogenic fatigue" who also improved with LSD‐assisted psychotherapy. In both cases, the ability of LSD to trigger repressed memories is noted.
Chandler et al (1960)^93^	LSD (route unspecified)	Starting dose of 25‐50 mcg, increasing by 25‐50 mcg per session until desired response.	1‐26; average of 6.2 per patient	Outstanding Improvement Marked Improvement Considerable Improvement Some Improvement Little or no change Slightly worse Definitely worse	Series included 1 patient with "conversion reaction." Details of presentation and treatment are not included, but the patient's response was classified as "some improvement."
Ling and Buckman (1960)^144^	IM LSD and methamphetamine (route unspecified)	Starting dose of LSD 40 mcg with escalation to "about 200" in very refractory patients. Average LSD dose 100 mcg	Limited details	Recovered Greatly Improved Moderately Improved Not Improved Worse	The series includes two patients with "conversion hysteria." No details of their presentations or treatment are provided, nor is there data available regarding response ‐‐ results were presented as aggregate response rates of all 50 patients in the study. Described in paragraph format is the case of a woman with sexual dysfunction and dyspareunia who, with LSD‐assisted psychotherapy, realized this to be connected with a history of childhood sexual abuse, and her symptoms eventually resolved.
Duche (1961)^95^	IM psilocybin	3‐9 mg	2	No scale provided. See remarks	Describes the case of a teenager with refractory functional dystonia that resolved following treatment with psilocybin. See "Case series and reports […]" section.
Heyder (1963)^97^	LSD (route unspecified)	300 mcg	3	No scale provided. See remarks	Describes the case of a welder with functional paralysis of the right upper extremity whose symptoms resolved following three sessions of LSD‐assisted psychotherapy. See "Case series and reports […]" section.
Ling and Buckman (1963)^99^	LSD (route unspecified) and methylphenidate	50‐75 mcg of LSD and 20‐30 mg of methylphenidate	3	No scale provided. See remarks.	Describes the case of a woman with functional urinary urgency treated with LSD and methylphenidate during three sessions of psychotherapy. In the weeks following her treatment, she experienced resolution of her symptoms. See Appendix A
Martin (1964)^101^	LSD (route unspecified)	Limited details	Limited details	No scale provided. See remarks	Briefly mentions a case of a 49 year old "paranoid depressive" who experienced total resolution of his conversion symptoms with LSD‐assisted psychotherapy only to have a relapse after learning that his girlfriend cheated on him.
Pos (1966)^103^	IM LSD	200‐1000 mcg; average dose 369 mcg	Average of 2.3 per patient	No scale provided. See remarks	Series includes 5 patients with "conversion syndrome": 1 with blindness, 1 with dysphagia, 2 with temper tantrums, and 1 with grand hysteria. The grand hysteria patient recovered, while outcomes are not given for the other patients except that the dysphagia patient was able to make a connection between her symptoms and repressed memories/emotions about her father. See "Case series and reports […]" section.
Baker (1967)^91^	IM LSD	Starting dose 100‐600 mcg	Limited details	Much Better Better Some Better Same Worse	Includes 3 patients labeled as having "conversion," 1 of whom was rated as much better and 2 as some better. One case of a "hysterical triplegic" is discussed in detail, the patient having improvement and ultimately resolution of symptoms with LSD‐assisted psychotherapy. See "Case series and reports […]" section.
Leuner (1967)^98^	LSD (route unspecified), psilocybin (route unspecified), CZ‐74^c^ (route unspecified)	Reports using "psycholytic" doses, which he defines as 30‐200 mcg of LSD or 3‐15 mg of psilocybin	Average of 26.7 per patient	Recovered Greatly Improved Moderately or Not improved	Includes 4 patients with "conversion‐hysteria," 1 who is rated as recovered, 2 as greatly improved, and 1 as moderately or not improved.
Martin (1967)^102^	LSD (route unspecified)	Limited details	6‐50 for hysteric patients	Recovered Greatly Improved Slightly Improved Not Improved	Includes "hysteria" as a diagnostic category and rates 6 of these patients as recovered and 3 as greatly improved. Details of the cases are not provided, but the author comments that after 6 years follow‐up, only one patient experienced slight relapse, which stabilized with psychotherapy.

^a^ 1‐acetyl LSD.^145^

^b^ 1‐methyl LSD.^146^

^c^ 4‐Hydroxy‐N,N diethyltryptamine.^147^

Variability in treatment protocol—as it pertains to dose, therapist involvement, and treatment setting—further limits the strength of conclusions that can be drawn from these prior cases. While higher (“psychedelic”) doses are typically used in modern studies, in the past, some therapists preferred using lower (“psycholytic”) doses.^2^, ^79^, ^104^ Regarding the dosing sessions themselves, an illuminating example can be found in a 1966 case series published by Robert Pos in which he reports the average duration of therapy sessions as being slightly over 2 hours^103^—this despite an average duration of LSD effects of 8.5 hours.^148^ Pos also mentions incorporating soft music into sessions only in the later phases of his study and admits to using soft restraints during some of the early sessions.^103^ Similarly, Edward Baker mentions fitting patients with Posey vests as part of his typical protocol.^91^ These practices are in stark contrast to modern standards in which patient psychological state and session environment (so‐called “set and setting”) are considered to be of primary importance in conducting safe and effective treatment sessions.^3^, ^79^, ^122^, ^124^, ^149^, ^150^

While the relative lack of details in many of these studies makes it difficult to draw conclusions from their results, there are at least four reports from the pre‐prohibition literature which clearly describe functional neurological symptoms being treated with psychedelic agents and are described below. Though not a FND per se, a fifth case of psychogenic urinary urgency treated with LSD is of relevance and is included in Appendix A.^99^

These cases provide potential insights into the therapeutic potential of psychedelic‐assisted therapy in functional disorders. All cases describe long‐standing, treatment‐refractory symptoms demonstrating rapid and sustained resolution following treatment with psychedelics. Cases 1 and 4 additionally provide some suggestion of a dose‐response relationship, something also noted by the aforementioned phantom limb patient.^138^ With the notable exception of case 4, all cases describe patients as developing insights into or change in connection with prior traumas, which may be of relevance to the therapeutic mechanism of these agents.

### 5.1 Case 1

Perhaps the most detailed description of a psychedelic substance being used to treat a FND is from a 1961 case report written by Didier‐Jacques Duché, a French child psychiatrist working out of La Salpêtrière Hospital in Paris. In part because the article is available only in French, his report will be described here in some detail.

The author describes the case of Annick B, a young girl treated for functional dystonia affecting her bilateral lower extremities. He reports that at age 12, following an episode of minor trauma, Annick began complaining of progressive left foot pain and difficulty walking. Despite a negative medical workup, she continued to walk with a limp through age 16 when, following a second episode of minor trauma, she developed pain in her other foot and associated increased difficulty ambulating, eventually developing what is described in the paper as an equinovarus deformity of both feet.

Annick is evaluated by several doctors who, “throughout various examinations, find no joint, muscular, bony, or neurological abnormalities,” and it is not until the following year when she is admitted to La Salpêtrière that she is diagnosed as having conversion disorder. The author offers the following description of the patient:

When examining the child for the first time, one is struck by the extent of her gait disturbance and deformity of her lower extremities. The feet are held in pronounced equinovarus, with only their outer edges supported by the floor. She walks with great difficulty, not by lifting the feet but instead sliding and waddling. There is an extremely intense muscle contracture apparently fixing the deformation. During the examination, the patient exaggerates this stiffness.

Despite endorsing a happy family environment, while undergoing narcoanalysis,^‡^ she admits that she is often awoken with the fear that a man is in her bedroom, and that this man resembles her father. Apart from this, her initial hospitalization course is described as “extremely mundane.” About this the author writes, “Different therapies are tried without being able to attribute one or another to a specific amelioration in symptoms with improvements being exceedingly gradual. Indeed, only towards the end of the second month does her gait return to normal.”

When seen initially in follow‐up, Annick's gait is described as quite normal, but it is said that she “keeps a behavior of the hysterical type as well as a certain anxiety.” The author goes on to say that after 3 months, she again develops progressive difficulty with gait, which is unsuccessfully treated with faradization.^§^ Eventually, the abnormalities of her lower extremities “resume their prior intensity,” at which point she is rehospitalized.

Despite 3 weeks in the hospital, Annick fails to demonstrate improvement, at which point her doctors decide to treat with psilocybin. She is first given a 3‐mg IM injection,^¶^ which results only in a vertiginous sensation and increased deep tendon reflexes on exam. The following day, however, she is given a 9‐mg injection after which she is described as passing through three stages: one of agitation, one of euphoria, and finally a depressive stage. It is during this last stage that she experiences an apparent breakthrough:

At the end of 2 hours, she declares that she must absolutely say certain things that she has never said before […]. She confesses then that her father and mother are inveterate alcoholics […] and that the arguments and the violence of which they are both guilty create an unbearable family life.

Indeed, the apparent inner liberation which accompanies these declarations is of greater interest than their very content. Nonetheless, in six years, this is the first time she gives details about her parents, a subject she had always avoided previously; even during the narco‐analyses, this problem had never been addressed.

In the following days, we very quickly witness the disappearance of the troubles and the resumption of a perfectly normal gait.

The author goes on to state that when Annick's case was last reviewed, 6 months after her hospitalization, there had been no recurrence of her symptoms.^95^

### 5.2 Case 2

In a 1963 article, Dietrich Heyder described the case of a 32‐year‐old man, who, after a job‐related welding accident resulting in second‐degree burns of his right hand, developed functional paralysis of that arm. He was referred to a psychiatrist 4 months after his accident, and a diagnosis of “conversion reaction” was made, though the patient denied emotional issues, and efforts by the therapist to uncover unresolved psychological conflict were unsuccessful even with the aid of hypnosis.

Though initially resistant, after 8 months of ongoing symptoms, the patient agreed to more aggressive treatment. While receiving sodium amytal infusion, he opened up about his experiences in combat in Korea and as a Chinese prisoner of war. He also detailed three events happening during this time period which, according to the author, “[fixated] his emotions on the right arm.” This included a story about a friend who lost his right arm in an explosion while substituting on patrol for the patient. The patient also reported that another friend had his right arm badly injured after being captured while trying to help the patient escape.

Following treatment, the patient regained mobility in his right arm but also began reporting severe muscular pains in his neck. After returning to work, the site of the welding accident, he experienced a relapse of his right arm symptoms, which did not respond to “psychotherapeutic interventions.” Over a year after his symptoms first began, LSD was tried as a last resort. A dose of 300 mcg was given three times over an 8‐day period. The author writes, “there was little communication and no change during the first two sessions, but a bout of motor hyperactivity, verbalization about past events, and hallucinations about being a windmill during the third LSD‐25 interview. The patient left the office with complete freedom of movement of the right arm.” The patient did not return to therapy, but telephone follow‐ups were conducted for over a year during which he reported sustained remission of his symptoms.^97^

### 5.3 Case 3

In a 1966 article, Robert Pos described his experiences treating 24 patients with LSD. Included in this is a case of “grand hysteria”,^∥^ a patient described as suffering for a number of years from “hallucinatory spells and seizures” which had been refractory to several interventions including intensive psychotherapy, hypnosis, ECT, and frequent hospitalizations. She was given three treatments with LSD (dose unknown) during which she “repetitively and perseveratingly states ‘I can say anything I like.’” Over the course of these sessions, she is described as developing increasing detachment to her prior traumas with subsequent and sustained remission of her seizures.^103^

### 5.4 Case 4

In a 1967 article, Edward Baker described the case of a refractory “hysterical triplegic” who had ongoing symptoms for a number of years following a hockey accident. The full details of his treatment are not provided, but the author states that “earlier LSD psychotherapeutic interview” resulted in recovery of two of his limbs, but that he continued to experience “mental amputation” of the leg at the mid‐thigh, which did not improve with escalating doses up to 1600 mcg. However, with a final dose of 2000 mcg, he regained full sensorimotor function within 10 minutes of injection after which, “he staggered down the ward, kissing whomever he saw, accepting congratulations.” His remission was sustained over 2 years of follow‐up. In addition to the tremendous doses of LSD used to treat this patient, the case is remarkable in that the patient was described as having no insight into his symptoms at the time of treatment or at any point during follow‐up.^91^

## 6 EVIDENCE AND ARGUMENTS AGAINST PSYCHEDELIC THERAPY IN FUNCTIONAL NEUROLOGICAL DISORDERS

In their 1963 book, “Lysergic Acid (LSD‐25) and Ritalin in the Treatment of Neurosis,” Ling and Buckman include a list of “unfavorable indications” for LSD psychotherapy, and listed among these is “gross hysteria, especially conversion hysteria”.^100^ These words of caution are repeated in Dr Buckman's 1967 article “Theoretical Aspects of LSD Therapy”.^155^ While the reasons for the recommendation to avoid LSD therapy in this patient population are not discussed in these works, in a published panel discussion from 1967, Buckman discusses his experience treating a “monosymptomatic conversion hysteria” patient who subsequently required hospitalization for several months for severe depression.^107^

In a 1954 article, Sandison et al report that the response of the conversion hysterics to LSD therapy was “not satisfactory” but also comment that these patients were seen early in the series, and that the amount of treatment given may have not been sufficient. Furthermore, their paper included only four patients of this type, one categorized as recovered, one as moderately improved, one as improved, and one who refused further treatment after a single session.^143^

Patients with functional disorders may also be prone to medication side effects.^156^ This raises some concerns about the tolerability of psychedelic‐assisted psychotherapy. In her 1959 manuscript, Margot Cutner writes the following:

It appears that under LSD, the tendency of the hysteric to evade attempts at consciously facing his problems, by producing or magnifying physical symptoms, becomes particularly obvious. Physical effects produced by the drug and largely ignored by some types of patients may become almost the only experience the hysteric realizes, overshadowing practically all psychological changes, and serving, in this way, as a particularly suitable means of expressing resistances. The writer found, for example, that headaches, feelings of nausea, restlessness, etc which frequently accompany early LSD reactions anyway, made LSD sessions almost useless with a number of hysterical patients, unless or until, through persistent analysis, the patient could accept his reactions as resistances and work through those. The writer seems to have found these reactions so consistently in hysterics that it almost appears possible to use LSD for diagnostic purposes [...] ^94^.

In addition to general concerns about side effects, it is possible that there could be unintended consequences to the sudden unmasking of repressed material. In thinking about functional symptoms from a teleologic perspective, one wonders whether expression of intrapsychic conflicts as a physical symptom may be an adaptive response and preferable to the emotional consequences of processing these conflicts on a conscious level. While such concerns would be in alignment with the generally slow and gradual methods of psychoanalytic psychotherapy, there is evidence in the literature to suggest that a more rapid approach can be effective. For example, in the aforementioned 2010 meta‐analysis of “drug interviews” for conversion disorder, in addition to suggestibility, abreaction was found to be predictive of recovery.^90^

## 7 CONCLUSIONS AND FUTURE DIRECTIONS

Given the relevance of nascent psychedelic neurobiological models to the pathophysiology of FNDs, and the critical lack of effective therapies for these disorders, detailed investigation into the relevance of psychedelics as an attestable treatment merits further investigation.

Nevertheless, we would advocate for a measured approach which seeks to avoid extrapolation of assumptions regarding dosing and tolerability from previous trials in other medical conditions. Specifically, while relatively high doses of oral psilocybin have been found to be well tolerated in a number of conditions (eg, addiction, OCD, and cancer‐related anxiety),^48^, ^49^, ^50^, ^52^, ^53^, ^54^ this does not necessarily imply that these doses would be well tolerated in patients with FNDs. An approach similar to the psilocybin for treatment‐resistant depression study published by Carhart‐Harris et al in 2016, in which a relatively modest dose of oral psilocybin (10 mg) was given during the initial treatment session before escalating to a high dose of psilocybin (25 mg) for the second treatment session,^56^ would allow for exploration of the dose‐response curve in FNDs and may provide more critical causal information related to 5‐HT_2A_ receptor modulation.

While all FNDs may be more similar than different in terms of underlying pathophysiology, we would recommend limiting initial studies to a subpopulation of patients with phenotypically similar presentations. Patients with nonparoxysmal functional motor symptoms may represent an ideal target population. This would not only allow for objective outcome measurement but also for introduction of, in addition to psychotherapy, physical therapy techniques during treatment sessions, which could increase the likelihood of a desired treatment response.

In addition to measuring clinical outcomes, and building on the preclinical work detailed in section 4, we would recommend pre‐ and post‐intervention functional imaging, with a specific focus on changes to within‐network functional connectivity of the DMN, as well as alterations in functional connectivity between the TPJ and sensorimotor cortex.

## ETHICS APPROVAL

Ethics approval was not required for this narrative review and perspective.

## DISCLOSURE

The authors confirm they have no relevant conflicts of interest to disclose.

## NOMENCLATURE OF TARGETS AND LIGANDS

Key protein targets and ligands in this article are hyperlinked to corresponding entries in http://www.guidetopharmacology.org, the common portal for data from the IUPHAR/BPS Guide to PHARMACOLOGY (Harding et al, 2018), and are permanently archived in the Concise Guide to PHARMACOLOGY 2019/20 (Alexander et al, 2019).^157^, ^158^

## PATIENT CONSENT

Patient consent is not applicable for this narrative review and perspective.

## Data Availability

Data sharing is not applicable to this submission as no new data or formal analysis of existing data were conducted for this article.

## ENDNOTES

* Abreaction: As defined in Merriam‐Webster, “abreaction is the expression and emotional discharge of unconscious material (such as a repressed idea or emotion) by verbalization especially in the presence of a therapist”.^106^

^†^ Mirror Therapy: First introduced in 1996 as a treatment for phantom pain, mirror therapy involves placement of a mirror in front of a patient in the sagittal plane, which creates the illusion that an amputated limb is still intact.^139^

^‡^ Narcoanalysis: Refers to psychoanalysis conducted while patients are under the influence of sedative‐hypnotic medications such as barbiturates.^151^

^§^ Faradization: refers to a form of electrotherapy in which brief faradic current is passed either throughout the body or to focal areas of dysfunction. These techniques were used to treat psychological conditions as well as physical ailments.^152^

^¶^ The bioavailability of oral psilocybin is approximately 50%.^153^

^∥^ Grand hysteria: A term coined by Jean‐Martin Charcot referring to psychogenic non‐epileptic spells resembling epileptic seizures with whole body convulsions.^154^

## Appendix A

### CASE 5

While not a functional neurological disorder per se, in a 1963 article, Thomas Ling and John Buckman describe the case of a 47 year old Scottish woman with frequent and severe urinary urgency which was limiting her daily activities. Her symptoms had onset suddenly 11 years prior when she found herself alone in a railway car with a young man. While he did not threaten her in any way, she found herself in a state of panic which was accompanied by a strong urge to urinate. This urge to urinate, which the patient refers to by the euphemism “penny spending,” became a frequent occurrence which persisted over the subsequent years, as did a worsened generalized anxiety—this despite counseling and attempts at treatment with “a variety of tranquilizers.”

In each of three sessions, she was given 50‐75 mcg of LSD with 20‐30 mg of methylphenidate. Over the course these sessions, both her urinary and anxiety symptoms significantly lessened, with continued improvement in the months that followed. The patient wrote the following in a progress report 4 months after completing treatment: My urge to ‘spend pennies’ has gone and I can now go out quite freely. It is a wonderful feeling of release. I still get thoughts about my old penny business, but I can laugh at it. The episode in the railway carriage is clear to me, and the understanding has made me feel so different about sex. The urge to ‘spend a penny’ was a substitute for my sexual feelings, which I always felt were wrong, and they were tied up together. For the first time in my life, I can let myself go in sex, and our marriage is much happier ^99^.
